# The Emerging Landscape of p53 Isoforms in Physiology, Cancer and Degenerative Diseases

**DOI:** 10.3390/ijms20246257

**Published:** 2019-12-11

**Authors:** Thineskrishna Anbarasan, Jean-Christophe Bourdon

**Affiliations:** School of Medicine, University of Dundee, Dundee DD1 9SY, UK; t.anbarasan@dundee.ac.uk

**Keywords:** p53, isoforms, cancer, p53 response

## Abstract

p53, first described four decades ago, is now established as a master regulator of cellular stress response, the “guardian of the genome”. p53 contributes to biological robustness by behaving in a cellular-context dependent manner, influenced by several factors (e.g., cell type, active signalling pathways, the type, extent and intensity of cellular damage, cell cycle stage, nutrient availability, immune function). The p53 isoforms regulate gene transcription and protein expression in response to the stimuli so that the cell response is precisely tuned to the cell signals and cell context. Twelve isoforms of p53 have been described in humans. In this review, we explore the interactions between p53 isoforms and other proteins contributing to their established cellular functions, which can be both tumour-suppressive and oncogenic in nature. Evidence of p53 isoform in human cancers is largely based on RT-qPCR expression studies, usually investigating a particular type of isoform. Beyond p53 isoform functions in cancer, it is implicated in neurodegeneration, embryological development, progeroid phenotype, inflammatory pathology, infections and tissue regeneration, which are described in this review.

## 1. Introduction

p53, first described four decades ago, is established as the “guardian of the genome” or master regulator of cellular damage response [1]. In response to a mutagenic or stress stimulus (e.g., nutrient deprivation) or changes in cell signalling and tissue homeostasis, p53 is activated, initiating different adaptive protein expression programs to maintain/restore tissue integrity and biological functions by triggering and coordinating different cell fate decisions in the diverse cell types composing the damaged organs. p53 induces and coordinates cell repair, cell survival, senescence, migration of immune, endothelial and progenitor cells, stem-cell renewal, differentiation and cell death depending on the cell type and function, the active signalling pathways, the type of damage, the cell cycle stage, nutrient availability, the immune response and the presence of pathogens [2,3,4,5,6,7].

p53 is at the hub of the cell signal pathways, being modified post-translationally and simultaneously by a plethora of diverse modifying enzymes (acetyl-transferase, deacetylase, methyltransferase, demethylase, ubiquitin-ligase, deubiquitinase, kinases, phosphatases, Poly-ADP ribose polymerase (PARP)) that are regulated by very different cell sensors and receptors [2,3,8]. In other words, p53, via regulating gene expression, integrates simultaneously many different cell signals and contribute to their translation into a cellular response precisely adapted to the type and context of the cell.

Adopting a dualistic perspective, the seemingly paradoxical effects of p53 become apparent, for instance, being able to transactivate both proapoptotic (*BAX*, *PUMA* and *NOXA*) and anti-apoptotic (*p21*, *14-3-3σ*) genes. Aptly described as “antagonistic bifunctionality”, this highlights the nature of p53 to mediate varied, at instances paradoxical cellular responses, influenced by the dynamic environment the cell is in [9,10]. Further supporting its dualistic nature, the cellular biochemical functions of p53, in addition to expected tumour suppressive effects, can have an oncogenic influence. The extent of the p53-mediated cellular functions still remains undefined. Furthermore, unlike conventional tumour suppressors, which are not expressed in human cancers due to non-sense mutation (stop) or gene deletion, p53 often undergoes missense mutations that do not entirely abolish all p53 biochemical activities [11,12]. It may explain why *TP53* mutations despite being the most frequently observed in human cancers, do not strictly correlate at the individual level to the patient’s clinical outcome [13,14,15].

The two distinct promoters of *TP53* gene (P1: upstream of exon1, and P2: within intron4), the alternative splicing and the alternative translation initiation sites of the different *TP53* mRNA lead to the co-expression of p53 proteins with different protein-interacting domains and activities (p53 isoforms) [16,17]. This review focuses on the current understanding of p53 isoforms and explores their established implications in both physiology and disease.

## 2. Generation of Human p53 Isoforms

The human *TP53* gene, as a transcription factor, comprises 13 exons (11 exons and 2 alternate spliced exons) located on chromosome 17p13.1 [18]. First observation of p53 splice variants was reported in the mid-1980s, but it was only 25 years after that *TP53* splice variants were discovered in various species, and their biological and clinical relevance established [19,20]. Alternative splicing of human TP53 intron 9 was subsequently described. Till date, twelve protein isoforms of p53 (p53α, p53β, p53γ, Δ40p53α, Δ40p53β, Δ40p53γ, Δ133p53α, Δ133p53β, Δ133p53γ, Δ160p53α, Δ160p53β, Δ160p53γ) encoded by 9 *TP53* mRNA transcripts, expressed differentially in tissues, have been described and characterized in humans [16,21,22,23,24].

p53 isoforms are generated as a result of a combination of the use of alternative promoters (P1 and P2), alternative splicing and alternative initiation of translation [22]. Canonical *TP53* transcription initiates at promoter P1. In humans, alternative splicing can occur to produce variants retaining intron 2 and intron9. Alternative splicing at intron 9 can produce mRNA variants with either inclusion of exon 9β or 9γ, giving rise to β and γ isoforms, respectively. Stop codons are present in exon 9β and 9γ; hence exons 10 and 11 remain untranslated for the β and γ *TP53* mRNA splice variants. The p53 mRNA transcribed from P1 promoter with spliced-out intron-2 can be translated from the first AUG (present in exon 2) generating p53α, p53β and p53γ proteins and from AUG40 due to an internal ribosome entry site in the 5′UTR from exon1-exon2 leading to the co-expression of Δ40p53α, Δ40p53β, Δ40p53γ isoforms [25,26]. From the *TP53* mRNA transcripts retaining the intron 2 (which contains stop codons), translation can only be initiated at AUG 40 producing thus only the Δ40p53 isoforms (Δ40p53α, Δ40p53β, Δ40p53γ isoforms) [16,22].

Transcription of *TP53* mRNA can initiate from internal promoter P2 located at intron 4. Translation of these transcripts from initiating codons 133 and 160 can generate Δ133p53 and Δ160p53 isoforms, respectively. Considering alternative splicing at intron 9, these transcripts give rise to isoforms (Δ133p53α, Δ133p53β, Δ133p53γ, Δ160p53α, Δ160p53β and Δ160p53γ isoforms) [22].

## 3. Conservation and Structure of p53 and Its Isoforms

The *TP53* gene has a dual gene structure that is conserved in several species such as zebrafish, drosophila, mouse and humans [22,27]. In drosophila, the *Dmp53* gene is the biological functional equivalent of the *TP53* gene. Two protein isoforms, DTAp53 and DΔNp53, homologous to Δ40p53 isoforms have been identified to be encoded by *Dmp53*. DTAp53 possesses the transactivation domain (TAD) defined by the highly conserved first 40 amino acids also detected in human p53α [16,28]. The zebrafish *Zp53* gene encodes Zp53α, Zp53β, ZΔNp53α and ZΔ113p5α isoforms. Other isoforms, associated with *Zp53* polymorphisms, have been detected in some zebrafish strains [28,29,30]. In mouse, the *TP53* homologue, *MsTP53* has till date been described to encode four isoforms, Mp53α, Mp53AS, MΔ41p53α and Mp53Ψ [31,32]. 

**Figure 1:**
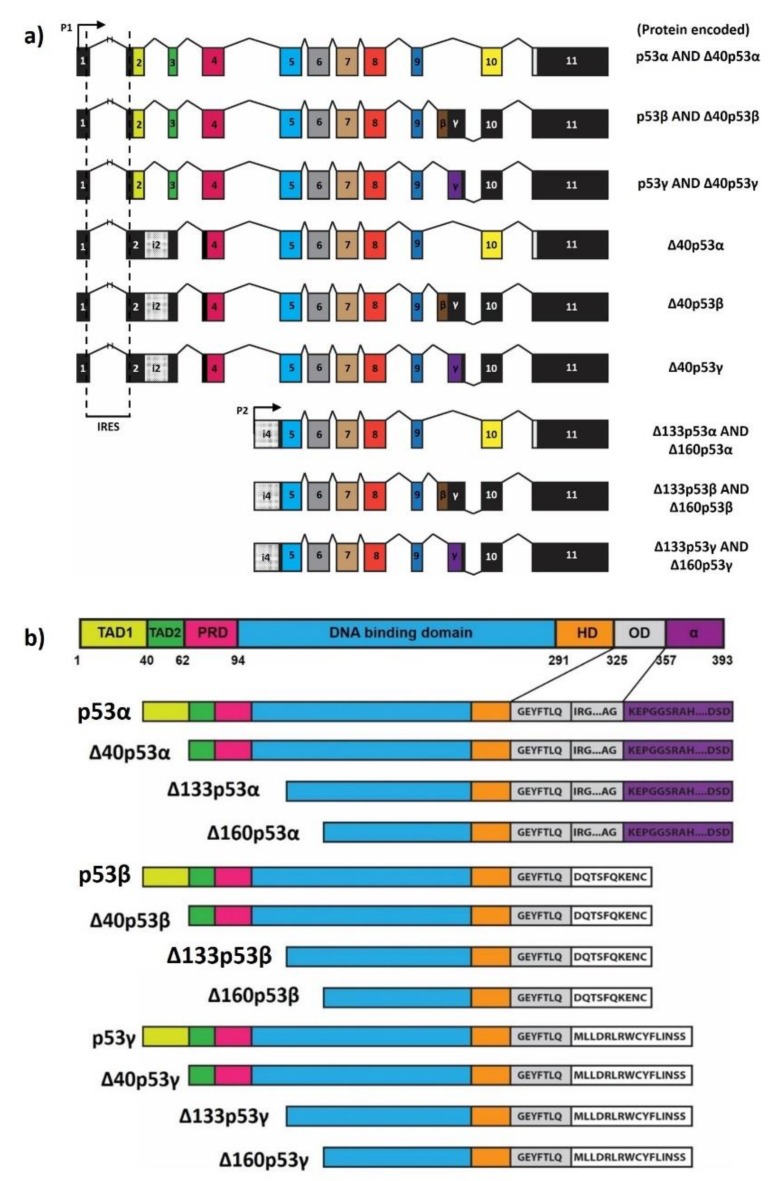
(**a**) p53 mRNAs. The *TP53* gene encodes 9 mRNA transcripts. (**b**) Functional domains of p53 and its isoforms. p53 contains seven functional domains which are the transactivation domain 1 (TAD1), the transactivation domain 2 (TAD2), the proline-rich domain (PRD), the DNA binding domain (DBD), the hinge domain (HD), the oligomerization domain (OD) and the negative regulation domain (α). At its N-terminus, lies an intrinsic disorder region (IDR) consisting of two acidic trans-activation domains (TAD), TAD1 (residues 1−39) and TAD2 (residues 40–61) and a proline-rich domain (PRD) (residues 62–93). This is followed by a DNA-binding domain (DBD) (residues 94–290) and a hinge domain (HD) (residues 291–324). At its carboxyl terminus, p53 comprises an oligomerization domain (OD) (residues 325–356) and a negative regulation domain (α) (residues 357–393).

p53α (canonical p53) is a DNA-sequence specific regulator of transcription orchestrating the expression of more than 3000 genes [33]. In its active state, p53α exists as a tetramer or stacking of tetramer on DNA [34]. p53α contains seven functional domains, as illustrated in Figure 1. The intrinsic disorder region (IDR), present in the N-terminal region, is an increasingly observed feature in proteins with influential roles within a signalling cascade [35]. IDRs allow a protein to form highly specific interactions with other functional proteins, albeit with low affinity [35,36]. The TADs comprised within the IDR is characterized by fleeting interactions with a wide range of proteins essential to the transcriptional activator function of p53 proteins. TADs can interact with components of the transcription machinery, chromatin modifiers, DNA metabolism proteins (e.g., PC4, HMGB1) and p53-modifying enzymes (e.g., MDM2, p300) [37]. From a functional perspective, TAD1 is integral for apoptosis and DNA damage-induced G1 arrest and both TAD 1/2 for mediating specific signalling pathways in the tumour suppressive response of p53 [38].

The proline-rich domain (PRD) joins TAD2 to the DNA-binding domain (DBD). It contains five PxxP motifs generating SH3 domain binding sites. Proline-rich regions can alter the 3D-structure of a protein though proline isomerisation (PIN1, cyclophilins) in the process regulating the orientation and angles of the interaction of its functional domains [39]. The length of the PRD and prevalence of specific docking sites within suggest an underlying function as a spacer or a scaffolding module necessary for the tumour suppressive role of p53 [39,40,41].

The DBD is the core domain of p53 that directly interact with DNA. It comprises of an immunoglobulin-like β-sandwich which provides the surface scaffolding for DNA binding [42]. The protein sequence of the p53 DBD contains numerous highly conserved histidine and cysteine whose coordination with Zn^2+^ or Mg^2+^, is integral for p53 conformation and DNA-binding activity [43,44]. p53 DBD interactions with its N-terminus can contribute to the stability of the p53 tetramer [45]. Mutations in DBD can cause conformational changes and/or alterations in specificity to p53-target DNA sequences. The propensity for carcinogenic mutations residing within this domain underscores its importance in the tumour suppressive and homeostatic functions of p53 [46]. Δ40p53 isoforms retain the complete DBD. In contrast, Δ133p53 isoforms lack a portion of first conserved cysteine box of the DBD, and Δ160p53 isoforms lack the entire first conserved cysteine box of the DBD. Despite truncations in the DBD, according to a recent publication, Δ133p53 and Δ160p53 isoforms can still have a stable 3D conformation [47].

The hinge domain (HD) is a short linker sequence of amino-acids between the DBD and the oligomerisation domain (OD). The HD offers structural flexibility for p53, allowing the binding of p53 response elements [48]. Germline mutations within the HD (R306P) have been associated with the loss of p53-mediated transcriptional activation of genes such as *BAX* [49]. Studies involving p53 devoid of the HD showed an inability to recognize consensus sequence, suggesting a possible role for the HD in the allosteric regulation of DNA binding [50].

The oligomerisation domain (aa 325–356), as its nomenclature suggests, is integral for the formation of p53 tetramers. The tetramerization process of p53 is best described as the formation of dimers between two primary dimers. The peptide presents within the oligomerisation domain links with another p53 monomer to form a primary dimer. This then assembles with another dimer to form a tetrameric structure that is stabilized by hydrophobic interactions between oligomerisation domains. Recently, lysine residues located within the oligomerisation domain, unessential for p53′s tetramerization capability, have been identified to selectively modulate p53-mediated apoptosis and cell-cycle arrest [51,52].

The extreme C-terminus of p53 containing the α domain is, like the TADs, an IDR. The extreme C-terminus domain, rich in positively charged amino acids (e.g., arginine, histidine, lysine) interacts non-specifically with negatively charged nucleic acids (RNA and DNA) [53]. Numerous proteins bind to the C-terminus domain, explaining why missense mutated p53 protein can still be biochemically and biologically active. The proximity of the C-terminus domain to the DNA-binding surfaces located within the DBD offers structural support for interactions with DNA. Additionally, the disordered α domain can form non-specific DNA interactions to allow the linear diffusion of p53α along DNA or to transfer its movement to another DNA molecule [54]. Most importantly, the α domain like the TAD undergoes a high degree of post-translational modifications (PTMs) which regulate protein degradation, tetramerisation, promoter selectivity and protein-interaction with the RNA-pol II transcriptional machinery [55].

## 4. p53 Isoforms Function in Concert

Various methodologies involving overexpression and/or siRNA knockdown in cell lines, or genetically modified animal models of p53 isoform expression have been developed and used to characterize the function of p53 isoforms [22,27]. The discovered biological functions of p53 isoforms include cell-cycle regulation, cell death, cellular senescence, inflammation, cellular invasion, antioxidant response, tissue regeneration and stem cell renewal and differentiation, which are outlined in Table 1. These cellular functions of p53 isoforms have been reported in the context of both malignant (including WT and mutant *TP53*) and non-malignant cells, originating from different tissues (e.g., skin, prostate, colon).

The biological functions of p53 isoforms are influenced by the expression of other isoforms. The retroviral expression of p53β in human fibroblasts inhibited cell proliferation and promoted cellular senescence via the upregulation of p21^WAF1^ and miR-34. However, this effect was not observed in *TP53*-null MDAH041 fibroblasts with hemizygous 1bp deletion at codon 184 (GAT-GAA, ter 244) [78], indicating that p53β does not induce senescence on its own but through cooperation with other isoforms produced by the *TP53* gene. Similarly, Δ133p53α isoforms’ pro-proliferative activity in WT *TP53* fibroblasts was not observed in *TP53*-null MDAH041 fibroblasts [16,56,57,65]. If some actions of p53 isoforms are only observed in the presence of other isoforms, it is of merit to investigate their biophysical interactions mediating cooperative behaviour.

**Figure 2:**
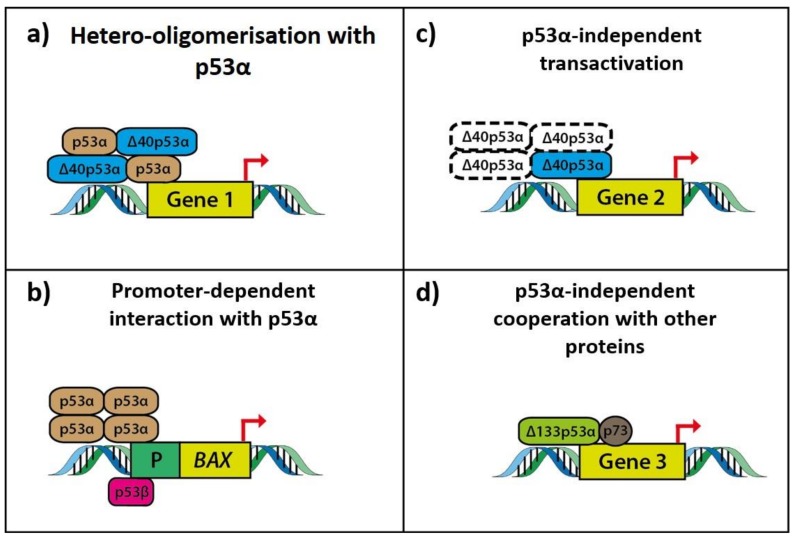
Schematic overview of p53 isoform interactions mediating transactivation. (**a**) p53 isoforms can form hetero-oligomers with p53α to mediate transactivation. For example, Δ40p53 α/p53α hetero-oligomers can modulate the transcriptional activity of promoters of IGF1-receptor and Nanog, thus controlling the switch from pluripotency to differentiation [64]. (**b**) p53 isoforms can transactivate target genes only in the presence of p53α. For example, p53β can indirectly interact with p53α in the presence of the *BAX* promoter DNA, modulating its promoter activity. Endogenous p53β binds to the *BAX* promoter in MCF7 cells; however, the exact p53β binding sequence on the *BAX* promoter remains to be elucidated [16]. (**c**) p53 isoforms can independently mediate transactivation. Δ40p53α can transactivate *BAX* and *GADD45* in p53-null cells; however, whether Δ40p53α mediates transactivation as an oligomeric complex remains unclear (represented by dotted lines) [24]. (**d**) p53 isoforms can mediate transactivation via cooperation with other proteins. p73 and Δ133p53α isoforms can cooperate in a p53-null environment to mediate DNA repair [72].

The interactions between p53 isoforms can be direct (e.g., formation of homo-oligomers or hetero-oligomers) or indirect (e.g., promoter-dependent oligomerisation) to convey and translate cell signalling in a defined cell response (Figure 2).

Although the canonical oligomerisation domain, required for the direct interaction and formation of hetero-oligomers with α-isoforms, is partially deleted in p53β isoforms, they can indirectly interact with p53α in the presence of the *BAX* promoter DNA (co-immunoprecipitation of p53β and p53α in presence of *BAX* promoter in H1299 cells co-transfected with p53α, p53β and Bax-luciferase reporter plasmid) modulating its promoter activity [16,79]. In contrast, Δ40p53α and Δ133p53α isoforms which retain the complete oligomerisation domain can directly interact with p53α (and probably other α-isoforms) to form distinct permutation of hetero-oligomers, which would differentially expose amino-acid domains to protein partners (i.e., Mdm2, MDMx) or cell protein machineries (i.e., transcriptional machinery, splicing machinery, microRNA machinery). Δ40p53α/p53α complexes can mediate biological responses by modulating the transcriptional activity of promoters of IGF1-receptor and Nanog, thus controlling the switch from pluripotency to differentiation [24,64,80]. In A375 melanoma cells, Δ40p53α/p53α complexes had modified promoter activity at *LRDD* and *CDKN1A* genes contributing to shifting cell-fate outcome in favour of apoptosis compared to cell-cycle arrest (upregulation of PIDD and downregulation of p21) despite exposure to γ-irradiation, an established trigger for p53-mediated DNA damage and cell cycle arrest [66].

Via direct hetero-oligomerisation, it has been reported that Δ133p53α can exert a dominant-negative effect on the apoptotic actions of p53α to favour p53 dependent DNA-repair and cell cycle progression [56]. Recently, von Muhlinen et al. coimmunoprecipitated p53α with overexpressed FLAG-tagged Δ133p53α and concomitantly observed decreased expression of p21 mRNA and miR-34a mRNA, consistent with the dominant-negative inhibition of p53α-mediated cell senescence [58]. However, the dominant-negative effect of Δ133p53α on p53α is not universally observed and therefore, is likely to be context and DNA-sequence dependent. In U2OS cells, the induction of Δ133p53α inhibited p53α-dependent apoptosis and G1 arrest but not p53α-dependent G2 arrest [81]. Similarly, instead of an exclusively dominant-negative relationship, the differential regulation (promoter and stress-dependent) of Δ113p53α on p53 was observed in zebrafish [66]. Factors contributing to this selective modulation of p53α’s transcriptional activity remain unclear. Furthermore, the functional significance of hetero-oligomers comprising of two or more types of p53 isoforms including/excluding p53α, has yet to be explored.

The effect of hetero-oligomerisation of isoforms can be dose-dependent, therefore influenced by the relative expression levels of the different isoforms. Δ40p53α at low levels increased p53α’s transactivation capacity, but at higher levels, inhibited p53α’s anti-proliferative effects [80]. Tetramers consisting of p53α and Δ40p53α isoforms were a more stable complex compared to a purely p53α complex [82]. One possible mechanism is that the Δ40p53α isoforms lack TAD1 precluding them from MDM2 mediated degradation [21,24]. The actions of MDM2 in the regulation of basal levels of p53α is in two ways. Firstly, MDM2 drives the translocation of p53α from the nucleus to the cytoplasm for proteasomal degradation. Alternatively, MDM2 can induce the ubiquitination of p53α to unmask the nuclear export signal located in the oligomerisation domain of p53α, making it more susceptible to nuclear export and degradation [83]. The p53 isoforms themselves have varied subcellular localization patterns possibly altering their susceptibility to MDM2 mediated degradation [16]. Δ40p53α isoforms, in particular, have been preferentially found in the nucleus [84]. Indeed, MDM2 can distinguish between p53 isoforms and differentially mediate their ubiquitination and degradation [85]. Hence, it is possible that the susceptibility of p53 hetero-oligomers to regulatory proteins such as MDM2 is dependent on the isoform composition adding a layer of permutation to the p53 response. 

Some p53 isoforms can bind to p53-response elements to induce the p53 mediated response. Δ40p53α can bind and transactivate independently of p53α some genes such as *MDM2, BAX* and *GADD45* genes [24]. Δ133p53α isoforms can in response to γ-radiation upregulate the transcription of DNA double-strand break repair genes including *RAD51*, *RAD52* and *LIG4* by binding to a novel type of p53-responsive element in their promoters independently of p53α [71]. Alternatively, p53 isoforms can, by directly interacting or cooperating with other proteins, exert their p53α independent cellular effects. Δ133p53β isoforms can interact with the anti-apoptotic protein, small GTPase RhoB, to negatively regulate RhoB activity inhibiting apoptosis [69]. Vascular smooth muscle proliferation has been found to be promoted by SRSF1, via the induction of Δ133p53a isoforms which in turn complexes with EGR1 to activate KLF5-p21 signalling [62]. In p53 deficient H1299 cells, the increased DNA double-strand break repair was associated with the overexpression of Δ133p53α isoforms and was reduced upon knockdown of p73. The changes in the levels of the latter alone had no effect of DNA double-strand break repair suggesting that p73 and Δ133p53α isoforms can cooperate in a p53α -null environment to upregulate the transcription of DNA double-strand break repair genes including *RAD51*, *RAD52* and *LIG4* [71,72].

**Figure 3:**
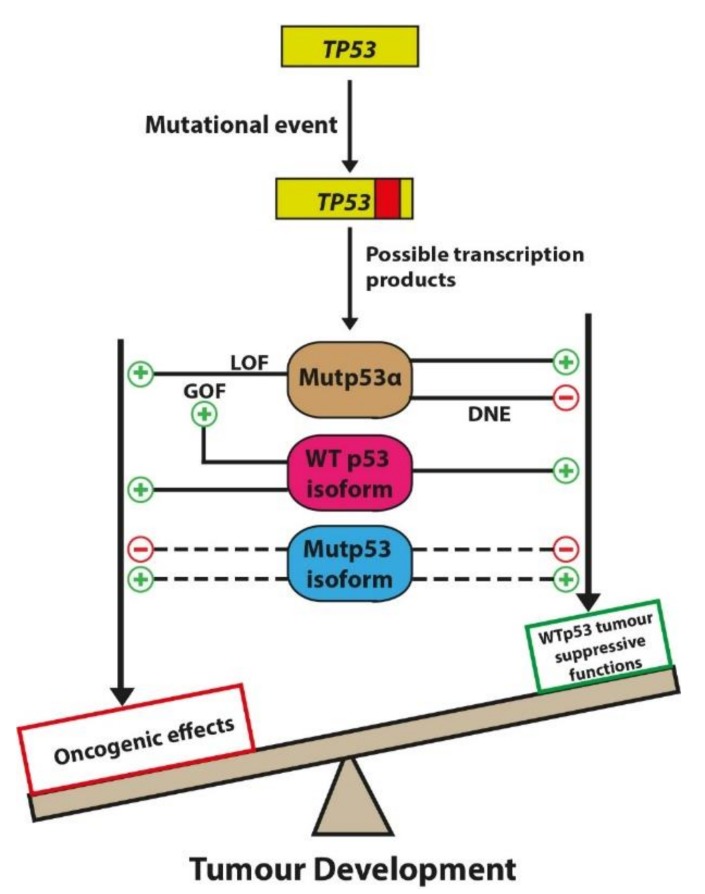
A *TP53* mutational event can result in the generation of Mutp53α, WTp53 and Mutp53 isoform. Mutp53α can mediate tumorigenesis via the following mechanisms: loss of WTp53 activity by dominant-negative effect (DNE) over the WTp53 allele. Alternatively, Mutp53α via gain of function (GOF) mutations promote oncogenic effects in a cell context-dependent manner [86]. WTp53 isoforms can contribute to tumour suppressive functions (e.g., Δ133p53α isoforms can coordinate with p73 to mediate DNA repair) and may also possess intrinsic oncogenic functions (e.g., Δ133p53β isoforms mediating angiogenesis). Preliminary evidence also suggests that WTp53 isoforms (Δ160p53α) can contribute to the GOF effects of Mutp53α [87]. The function of Mutp53 isoform in tumorigenesis remains unclear (represented by dotted lines) but, could theoretically mediate both oncogenic and tumour suppressive effects. Together this provides an overview of the possible downstream p53 isoform effects following a *TP53* mutational event.

Next, the question emerges as to which p53 mediated biological activities are p53α-dependent and which ones are instead, influenced independently of p53α by the other isoforms. This has significant biological implications, as for example, a mutation upstream of codon 133, excluding promoter and splice site mutations, will unalter Δ133p53α mediated DNA repair but may perturb other p53-mediated functions reliant on WT upstream domains (i.e., apoptosis). Hence, it is conceivable that upon a *TP53* mutational event and the consequential abrogation of p53α-response, unaffected WT p53 isoforms can continue to exert their p53α-independent cellular effects influencing the severity of the consequential carcinogenic process (Figure 3).

p53 isoforms were observed to exhibit paradoxical effects, such as promoting both cellular proliferation and apoptosis, albeit in different cellular contexts. For example, p53β induces cellular senescence in normal human fibroblasts, CD8+ T lymphocytes and MCF7 cells but promoted cellular proliferation in the latter upon treatment with TG003 [65]. To guard cellular homeostasis, spatiotemporal interactions between p53 isoforms (e.g., hetero-oligomerisation), can mediate a non-binary, robust cellular response algorithm (to a physiological or external stimulus; e.g., DNA damage, virus) which remains inadequately understood, contributing to the perceived paradox. Therefore, it would be an oversimplification to merely classify individual p53 isoforms as either exclusively being tumour suppressors or oncogenes [57,65].

In replicative senescent cells, the expression of p53β was increased and Δ133p53α decreased in comparison to non-senescent cells [56]. The significance of protein isoform imbalance contributing to pathology has been previously recognized, most notably in tauopathies. A shift in the normal balance of tau isoforms (3R and 4R) in the human brain has been associated with abnormal neuronal firing and cognitive impairment. Modulating the tau isoform imbalance with RNA reprogramming (trans-splicing strategy) was associated with reduced accumulation of pathological tau and is therefore regarded as a promising therapeutic strategy for neurodegenerative diseases and may possess similar therapeutic value for pathology associated with p53 isoform imbalance [88]. Investigating therapeutics at the protein level, Lei at al. showed evidence of p53 isoform modulation via introducing a p53 peptide (107–129) to stabilise and shift the conformation of Δ133p53β to that of WT p53 [47]. However, whether the functional effects of Δ133p53β have been altered remain to be studied. This strategy albeit preliminary may be of value in malignant pathology with increased expression of Δ133p53β, associated with cancer invasiveness (discussed in the next section).

## 5. p53 Isoforms in Cancer

Dysregulation of p53 isoform co-expression may alter, without abolishing, the predicted p53 response, therefore driving carcinogenesis while simultaneously conferring sensitivity to a type of cancer treatment. Several studies have attempted to investigate the expression of p53 isoforms in both cancerous and normal tissues, largely utilizing RT-qPCR based assays. From these studies, it is apparent that p53 isoform expression is not random (Table 2). In breast tumours, Gadea et al. reported that Δ133p53α was co-expressed with other isoforms including Δ133p53γ and Δ133p53β [76]. In head and neck squamous cell cancer, p53β was found to be consistently overexpressed in almost all samples investigated and Δ133p53 isoforms were overexpressed in non-small cell lung cancer compared to adjacent normal tissue [89,90]. The non-random, isoform co-expression was also observed in premalignant tissue, where p53β was elevated and Δ133p53α reduced in colonic adenoma in contrast to normal or non-adenoma tissue [56].

Up to now, clinical studies have largely investigated one isoform at a time, however, it is becoming clear that the p53 isoforms are co-expressed and work in concert to define cellular responses. Therefore, to better understand p53 isoform activities, future studies evaluating the relationship between p53 isoform expression and clinical outcomes should adopt investigating the co-expression of the different p53 isoforms in combinations.

The increased expression of p53β has been associated with improved disease-free survival in breast and clear cell renal cell carcinoma [93,104]. In cancer cell lines and normal fibroblasts endogenously co-expressing p53 isoforms, the overexpression of p53β induces apoptosis and cell senescence via the upregulation of genes such as *BAX* and *p21*/*miR34* in a p53-dependent manner [56]. The tumour suppressive effects observed in cell studies may explain the improved cancer outcomes associated with the expression of p53β. The expression of the alternate C-terminal variant p53γ, with other isoforms, is predominantly associated with reduced risk of cancer progression as reported in breast and uterine squamous cell carcinoma [94,106]. 

The expression of Δ40p53α is higher in tumours than normal tissue in both glioblastoma and breast cancer [93,101]. The clinically more aggressive triple-negative breast cancer subtype is associated with increased Δ40p53α expression [93]. Contrastingly, in mucinous ovarian cancer, the expression of Δ40p53α is associated with improved disease-free survival [97]. Taking this together, the biological relevance of Δ40p53α is evident, however, it is not possible to attribute to it an absolute oncogenic or tumour suppressive role since its activity is dependent on cell function, cell context and the co-expressed driver oncogenes.

The overexpression of Δ133p53α in tumour tissue has been reported in cholangiocarcinoma, lung, colon and ovarian cancers [56,90,98,100]. In lung cancer tissue, p21 expression was reduced whereas Δ133p53α was overexpressed, however, this inverse relationship did not reach statistical significance possibly owing to the small sample size [90]. Further evidence of this relationship is observed in breast cancer, where Δ133p53α inhibits the ability of p68 to induce p53-dependent transcription from the *p21* promoter [91]. This suggests that tumour cells with Δ133p53α expression could have reduced ability to undergo p21 mediated cell-cycle arrest. Increased Δ133p53α isoform expression, in the context of WT *TP53*, has been associated with poorer disease-free survival in patients with colorectal cancer. A possible mechanism explaining this association is Δ133p53α isoform mediated tumour cell invasion via increased IL-6 expression activating JAK-STAT3 and RhoA-ROCK pathways [77]. Interestingly, in advanced serous ovarian cancer tissue with mutant *TP53* gene, Δ133p53α expression was associated with improved disease-free survival and overall survival, providing preliminary evidence that the mutational status of *TP53* can influence the association between p53 isoform expression and patients’ clinical outcome.

In breast cancer, Δ133p53β expression was significantly associated with an increased risk of cancer recurrence and poorer overall survival [76,92]. In HCT116 cells, Δ133p53β expression was found to promote the acquisition of an amoeboid-like phenotype associated with epithelial mesenchymal transition and cell invasiveness [76]. Recently Kazantseva et al. showed that the expression of Δ133p53β, which was increased in glioblastoma tissues with WT p53, promoted an immunosuppressive tumour microenvironment by increasing CCL2 expression and subsequent CD163 macrophage infiltration [102]. Taken together, it is becoming apparent that Δ133p53β co-expression with other p53 isoforms could be associated with poorer cancer outcomes.

The expression and cellular effects of p53 isoforms can be influenced by the mutational status of *TP53*. Therefore, it is recommended that the p53 isoform expression is reported with respect to the mutational status of *TP53*, to establish more precise correlations with clinical outcomes [109,110]. However, stating whether p53 is WT or mutant may not suffice as it is becoming apparent that the mutational landscape of p53 is highly heterogeneous and the various p53 mutants are not equal in their cellular and phenotypical effects [111,112]. For example, Li Fraumeni syndrome (LFS) patients with specific p53 DBD mutations (associated with impaired anti-proliferative functions in H1299 cultured cells) have tumour incidences at an earlier age compared to LFS patients harbouring germline *TP53* mutations retaining anti-proliferative functions [112].

A feasible step towards representing p53 isoform expression in relation to the heterogeneous p53 mutations would be to adopt a functional-domain oriented approach by classifying p53 mutants as follows: TAD, oligomerisation domain and DBD mutants [113]. Studies investigating mutations (missense mutations, insertions and deletions) within the TAD of p53 revealed that these mutations often introduce a stop codon upstream of the ATG codon in exon 4, disrupting the expression of p53α. These mutants, classified as TAD mutants of p53, consequentially express all p53 isoforms except p53α, p53β and p53γ. As these mutants still possess the ability to partially transactivate p53-dependent genes, including pro-apoptotic genes, TAD mutants could be generally more responsive to treatment and associated with a better prognosis than DBD mutants [113,114]. The impact of a mutation in the exons coding for the oligomerisation domain and DBD on the expression of p53 isoforms is less clearly defined as compared to that of TAD mutants.

Select p53 isoforms are often studied in isolation, leaving the implications of any potential changes in other isoforms unclear. As a type of p53 isoform is never expressed exclusively as a single p53 protein in cancer or normal cells, characterisation of their combined activities is required to recapitulate physiological expression. Nutthasirikul et al. identified that the Δ133p53α/p53α ratio was an independent predictor of poor prognosis in intrahepatic cholangiocarcinoma [100]. A lower Δ40p53α:p53α ratio was associated with reduced cancer progression in breast cancer [95]. With consistent associations being observed (e.g., p53β expression correlating with a better prognosis), a concerted effort to decipher how the combined co-expression of various isoforms and type of p53 mutation alter cancer prognosis and survival is required.

## 6. Implications of p53 Isoforms Beyond Cancer

The role of p53 in embryological development has been a perplexing puzzle that has not yet been fully understood, and more than one type of p53 isoform has been implicated. p53 null mouse, zebrafish and drosophila models have been developed showing different embryogenic abnormalities and also possess a phenotype characterized by an increased incidence of cancers at an earlier age [115,116,117,118,119]. When p53 was overexpressed or hyperactivated in transgenic animal models, embryonic defects were observed with consequential phenotype influenced by the magnitude and spatiotemporal pattern of p53 activation [120]. In mouse models with *TP53* expressing Δ122p53, isoforms (equivalent to human Δ133p53) were associated with abnormalities in embryogenesis, with a predilection for female embryos. These include exencephaly, ophthalmoplegia, ectopic vertebrae and foetal reabsorption [31]. In zebrafish, the loss of function mutation in the *Def* gene impaired digestive organ growth and induced the expression of the ZΔ113p53α isoform [115]. This suggests that in the zebrafish ZΔ113p53α isoforms are negatively regulated by the *Def* gene during embryogenesis to avoid dysregulation of organogenesis. Taken together, these indicate that the ZΔ113p53α isoforms could have a role in normal embryogenesis and whether this is p53α dependent remains unclear [30].

Transgenic mice ectopically expressing Δ40p53α isoform in the presence of WT p53 had a progeroid phenotype consistent with accelerated ageing (reduced bone mineral density and loss of fertility). The disrupted Δ40p53α:p53α isoform ratio in these mice was associated with the upregulation of IGF-1 signalling [59]. Δ40p53α isoforms functionally inactivate PTEN which regulates IGF signal transduction to Akt, thus dysregulating IGF-1 signalling and consequentially promoting cellular senescence and reduced proliferation. Glucose homeostasis was also adversely affected in Δ40p53α transgenic mice, characterized by early-onset hyperinsulinemia and glucose intolerance, resulting in hyperglycaemia and impaired proliferation of β-cells in the pancreas. Abnormal β-cell growth homeostasis could be a result of dysregulated Akt regulation of CDK4, cyclin D1 and cyclin D2 via the effects Δ40p53α isoforms on IGF1 signalling [121]. In the rare progeria disease, Hutchinson–Gilford progeria syndrome (HGPS), the premature ageing phenotype has been associated with the overexpression of p53β and the reduction of Δ133p53α isoforms. Further analysis in proliferative HGPS fibroblasts with the depletion of Δ133p53α isoforms was associated with increased mRNA expression of p53 target genes associated with cellular senescence (*p21/CDKN1A*), senescence-associated secretory phenotype (SASP) and pro-inflammatory cytokines (IL-6 and IL-8), hence explaining the progeria phenotype [58].

Ageing is a direct risk factor for neurodegenerative conditions such as Alzheimer’s disease. In a mouse model, the overexpression of Δ40p53α isoform, associated with an accelerated ageing phenotype, conferred dysregulated tau phosphorylation and cognitive dysfunction. The mouse Δ40p53α isoform was observed to promote tau phosphorylation via binding to promoters and inducing transcription of several kinases (Dyrk1A, GSK3β, Cdk5, p35, and p39) [58]. Overexpression of p53β in WT *TP53* expressing cells, consistent with investigations from cancer cell lines, induced senescence in astrocytes. In a state of senescence, cells secrete a host of cytokines, proteases and growth factors that alter the tissue microenvironment [122]. However, when astrocytes are in a state of senescence, the resulting SASP induces neurotoxicity which can be observed with overexpression of p53β or the knockdown of Δ133p53α isoforms. Indeed, in the brain tissue of patients with Alzheimer’s disease and amyotrophic lateral sclerosis, p53β was upregulated and Δ133p53α isoforms downregulated compared to age-matched normal brain tissue [123]. More recently, in response to irradiation, Δ133p53α isoforms were observed to have a protective role against radiation-induced brain injury (risk of reduced cognitive and vocational ability), via inhibiting senescence and preventing astrocyte-derived neuroinflammation in human astrocytes [123].

p53 isoforms can mediate a chronic pro-inflammatory state similar to disease states observed in autoimmune inflammatory arthropathies and vasculitis. Serum analysis of mice expressing Δ122p53 isoforms revealed increased expression of inflammation-associated cytokines such as IFNγ, TNFα, IL6, CCL2 and GM-CSF. When IL-6 was depleted in these mice, fewer tumours were observed and mediators of the JAK-STAT signalling pathway were downregulated, supporting a role for p53 isoform induced inflammation in carcinogenesis [31].

Bacterial and viral exposure is a recognized form of cell stress. Viral infections are especially well-established deregulators of the p53 pathway (e.g., HPV, the Epstein Barr virus or influenza virus). Helicobacter pylori can upregulate the expression of Δ133p53 isoforms in vivo via activation of transcription factors cFOS/cJun ultimately deregulating p53α [123]. This reduced p53α mediated stress response signalling, allowing continued progression of helicobacter pylori-associated pathogenesis and eventually tumorigenesis.

In Drosophila, p53 isoforms can mediate tissue regeneration by influencing apoptosis-induced proliferation (AiP) which is a phenomenon characterized by the release of mitogens such as hedgehog (Hh) and wingless following stress stimulus to promote the proliferation of adjacent cells to restore organ integrity and function [22,124,125]. In mice, the splice Mp53Ψ isoform is upregulated in stem cells in a tissue-specific manner following a damaging event. In liver tissue, CCL4-induced lesions regress in size following expression of Mp53Ψ, supporting its role in tissue regeneration. In humans, however, endogenous Mp53Ψ has only been characterized in the HOP62 cancer cell line which possesses a mutation in the acceptor splice site of *TP53* intron 6 [32]. This, therefore, associates *TP53* splice site mutations with oncogenic functions. Taken together, much of the evidence on p53 isoform role in tissue regenerations is convincing albeit limited to animal models.

## 7. Detecting p53 Isoforms

RT-qPCR has been the most widely used and reliable technique to investigate p53 isoform expression. Alternatively, the approach to detect p53 isoform expression at the transcriptome level via analysis of RNA-sequence data from large cancer repositories such as The Cancer Genome Atlas (TCGA) proves challenging with the available bioinformatic platforms (RSEM method) having poor sensitivity for detecting low abundance splice variants [126]. The challenge with deriving conclusions based on the level of p53 mRNA variants is that their expression levels do not always correlate with the protein expression level of the corresponding isoforms [22]. Several antibodies have been developed to investigate p53 isoform expression at the protein level using Western blotting, immunohistochemistry (IHC) or immunofluorescence [22]. The use of IHC remains a common technique to investigate *TP53* mutational status in routine pathology [127]. Therefore, given the significance of p53 isoforms in multiple biological processes, it would be a logical endeavour to investigate p53 isoform expression and their spatiotemporal distribution in normal, precancerous and cancer tissues by IHC. Several groups have attempted to identify p53 isoforms with IHC with varying degrees of success [92,102,128]. Significant limitations experienced include the paucity of p53 isoform-specific antibodies and weak signal intensity. The latter is an expected challenge in attempting to visualize p53 isoforms, including p53α, via IHC as they are not as abundantly expressed as structural proteins such as actin, vimentin and cytokeratin. Additionally, as p53α is notorious for extensive PTMs, one would expect p53 isoforms to also be similarly modified, thus potentially affecting the target epitope of the isoform-specific antibodies. Other methods to study isoform expression in situ include RNAscope in situ hybridisation which has been employed to study Δ133p53β expression in formalin-fixed paraffin-embedded glioblastoma tissue [102]. Targeted proteomics with liquid chromatography-tandem mass spectrometry (LC-MS/MS) is emerging as a reliable method to quantify p53 isoform expression at the protein level. For example, Jiang et al. combined molecularly imprinted polymers and LC-MS/MS to reliably quantify C-terminus p53 isoform variants in several breast cancer cell lines [129].

## 8. Concluding Remarks

Based on the accumulated evidence, it is gradually becoming evident that the dysregulation of p53 isoform balance is biologically relevant not only in the pathogenesis of cancer but also in neurodegenerative, inflammatory, infection and progeria associated diseases. Investigating p53 isoforms offers a new vantage point to analyse and unravel the multiple biological functions of p53, which have been elusive to therapeutic targeting in the last few decades. Numerous questions remain; hence, a rigorous, concerted effort is essential to (1) develop more p53 isoform-specific antibodies with appreciation of PTMs; (2) interrogate the mechanism underpinning the shift in isoform balance in response to a particular cell context or stimulus; and (3) design clinical studies which investigate the co-expression of p53 isoforms with outcomes. These are key steps towards deciphering the role of p53 isoforms in both pathology and physiology and determine how clinicians can manipulate the isoforms’ activities to rationally trigger the appropriate tumour response on patient clinical outcomes.

## Figures and Tables

**Table 1:** p53 isoforms collectively classified according to reported biomolecular functions. Respective cell/animal models used to investigate isoform function is presented in association with observed alteration in protein/gene expression/activity following p53 isoform manipulation. Implicated genes are in italics.

Cellular Function Involving p53 Isoforms	Cell Line/Model(s) Studied	Altered Expression or Activity	Reference
Cell cycle regulation	MRC-5, WI-38	*miR-34a,* *CDKN1A* *, PAI-1, IGFBP7, MMP3, BUB1, CDC20*	[56]
	CD8+ T lymphocytes	CD62L, PD-1, LAG-3, *IL-6, IL-8, SRSF3, CD28, CD57*	[57]
	HGPS fibroblasts	STUB1, *CDKN1A, IL-6, IL-8, SRSF3*	[58]
	Transgenic mice model	IGF-1R, IGF-1, Gadd45, PTEN, *MDM2,* *CDKN1A, IGFBP-3*	[59]
	Transgenic mice model	*p66Shc, G2-M genes*	[60]
	Transgenic mice model	*MDM2, CDKN1A*	[61]
	HASMCs	EGR1, *SRSF1, KLF5, p21*	[62]
	Human neonatal foreskin and normal prostate tissue	*CDKN1A, PUMA, NOXA, hTERT*	[63]
	129/SvJ ESCs	*CDKN1A, MDM2*	[64]
Apoptosis	MCF7	SRSF1, p21, BAX	[65]
	H1299	BAX, p21^WAF1^	[24]
	A375 melanoma cells	*CDKN1A, PIDD*	[66]
	Zebrafish model	*CDKN1A, MDM2, BCL2L*	[67]
	Zebrafish model	*CDKN1A, BAX, MDM2, BCL2L*	[68]
	H1299	*BAX*	[16]
	HCT116, SW480, LoVo, SW620, Colo205	RhoB	[69]
	Transgenic mice model	ANXA5, TPT1	[70]
	Transgenic mice model	*BIRC5, TRAF1*	[61]
	Human neonatal foreskin and normal prostate tissue	*BAX*	[63]
DNA repair	HGPS fibroblasts	*RAD51*	[58]
	QSG-7701, Zebrafish model	*RAD51, LIG4, RAD52*	[71]
	Transgenic mice model	VCP	[70]
	Saos2, HCT116, H1299	p73, *RAD51, LIG4, RAD52*	[72]
Inflammatory response	Transgenic mice model, SaOS2	*STAT1, STAT2, OAS1g, IFI47, IFIT2, CXCL10*	[73]
	Transgenic mice model	Alpha-enolase, TNF-alpha, CCT5, 14-3-3, ALDH2	[70]
	Transgenic mice model	IL-6, IFN-g, TNF-alpha, IL-3, IL5, *STAT1, JUNB*	[61]
Autophagy	HCT116, H1299	p-PKR, p-elF2α, *DRAM*	[74]
Pluripotency	MCF7	*SOX2, OCT3/4, NANOG*	[75]
	129/SvJ ESCs	OCT4, GATA-4, *NANOG, IGF-1R*	[64]
Cellular invasion	MDA-MB-231, D3H2LN, MCF7, LoVo, SW480, SW620, Colo205, HCT116	E-cadherin, β1-integrin	[76]
	Transgenic mice model	*ITGB7, VCAM1*	[61]
	Transgenic mice model, HCT116	RhoA, *IL-6*	[77]

**Table 2:** Summary of p53 isoform expression and associated clinicopathologic outcomes in human cancers. *n* refers to the number of patients from whom the samples analysed were obtained from each study. Cholangiocarcinoma (CCA); glioblastoma (GBM); renal cell carcinoma (RCC); uterine squamous cell carcinoma (USC); acute myeloid leukaemia (AML); squamous cell carcinoma of head and neck (SCCHN); endometrial carcinoma (EC).

Cancer	Isoforms Studied	N	Summary of Key Results	References
Breast	Δ133p53α, Δ133p53β/γ	147	Inverse association in expression of Δ133p53β mRNA with p68 protein.	[91]
	p53β, Δ40p53a, Δ133p53β	47	Δ133p53β isoform increased in invasive breast carcinomas compared to non-invasive cases.	[92]
	p53β/γ, Δ40p53α, Δ133p53α	148	Δ40p53 was increased in tumour breast tissue and associated with aggressive subtype. p53β expression was associated with poorer disease-free survival.	[93]
	Δ133p53α/β/γ	273	Δ133p53β reduced in HER2 positive tumours and is associated with poorer disease-free and overall survival	[76]
	p53β/γ	127	Mutant p53 breast tumour-expressing p53γ isoform had improved disease-free survival. p53β was associated with tumour oestrogen receptor (ER) expression	[94]
	Δ40p53α	139	Reduced Δ40p53α:p53 ratio associated with improved disease-free survival.	[95]
Ovarian	Δ40p53α, Δ133p53α	169	No difference in p53 isoform expression between stage I and III ovarian cancer.	[96]
	Δ40p53α, Δ133p53α	166	Δ40p53α expression associated with improved disease-free survival in patients with mucinous ovarian cancer with WT *TP53*. Increased Δ133p53 expression in endometroid ovarian cancer.	[97]
	Δ40p53α, Δ133p53α	154	Δ133p53 expression associated with improved disease-free and overall survival in p53 mutant serous ovarian cancer. Increased Δ40p53 expression associated with improved disease-free survival but not overall survival in p53 WT serous ovarian cancer.	[98]
	p53α/β/γ, Δ133p53α	69	No difference in isoform expression between chemo responders and non-chemo responders. Increased Δ133p53α expression significantly associated with improved overall survival and borderline significance for improved disease-free survival.	[99]
Colon	p53β, Δ133p53α	29	Colon adenoma tissues expressed elevated p53β and reduced Δ133p53α expression compared with non-adenoma and normal colon tissue. Δ133p53 isoform expression was significantly higher in carcinoma tissue	[56]
	Δ133p53α/β	35	Increased Δ133p53α expression associated with poorer disease-free survival.	[77]
CCA	Δ133p53α	48	Increased Δ133p53 and Δ133p53/p53a expression associated with a poorer overall survival.	[100]
GBM	Δ40p53α, p53β	17	Δ40p53α was observed in glioblastoma tissue which was not detected in non-tumour cerebral cortex.	[101]
	p53β, Δ40p53α, Δ133p53α	89	Δ133p53β expression increased on a wild-type *TP53* background in glioblastoma.	[102]
RCC	p53β/γ, Δ133p53α/β/γ	41	p53β mRNA was overexpressed in tumour samples and correlated with tumour stage.	[103]
	p53β/γ	268	p53β expression was associated with improved disease-free and overall survival in p53 mutant patients.	[104]
	p53α, Δ40p53α, Δ133p53α	41	Expression of p53 p53α, Δ40p53α, Δ133p53α was increased in mutant *TP53* RCC compared to WT *TP53* RCC.	[105]
EC	p53β/γ, Δ40p53α, Δ133p53α	37	Increased p53γ expression is associated with poorer disease-free survival	[106]
AML	p53β/γ	68	p53β and p53γ expression correlated with mutated NPM1, a marker of improved overall survival.	[107]
SCCHN	p53β/γ, Δ133p53α/β/γ	21	p53β/γ, Δ133p53α/β/γ were detected in tumour tissue.	[89]
Lung	Δ133p53α	17	Overexpression of Δ133p53 mRNA was observed in cancerous tissue as compared to adjacent non-cancerous tissue.	[90]
Melanoma	p53α/β/γ, Δ40p53α/β/γ, Δ133p53α/β/γ, Δ160p53α	38	In tumour tissue, Δ40p53β expression was reduced, whereas Δ133p53α and Δ160p53α expression was increased. Reduced p53β expression or increased Δ133p53β and p53α mRNA expression were associated with poorer overall survival.	[108]

## Abbreviations

IRES	Internal ribosome entry site
TAD	Transactivation domain
IDR	Intrinsic disorder region
DBD	DNA-binding domain
PTM	Post-translational modifications
HGPS	Hutchinson-Gilford Progeria Syndrome
SASP	Senescence associated secretory phenotype
AIP	Apoptosis-induced proliferation
Hh	Hedgehog
IHC	Immunohistochemistry
PRD	Proline-rich domain
HD	Hinge domain
LFS	Li Fraumeni Syndrome
AML	Acute myeloid leukaemia
PARP	Poly-ADP ribose polymerase
